# 2-(3-Nitro­phen­yl)-4,5-diphenyl-1*H*-imidazol-3-ium nitrate

**DOI:** 10.1107/S1600536809023599

**Published:** 2009-06-24

**Authors:** Yi Zhang

**Affiliations:** a Department of Physics, Southeast University, Nanjing 210096, People’s Republic of China

## Abstract

In the cation of the title compound, C_21_H_16_N_3_O_2_^+^·NO_3_^−^, the N atom in the 3-position of the imidazole ring is protonated. The three pendant aromatic rings are twisted from the plane of the imidazolium ring by dihedral angles of 22.75 (1), 79.63 (1) and 29.65 (1)°. In the crystal structure, N—H⋯O hydrogen bonds link the mol­ecules, forming an infinite one-dimensional chain parallel to the *b* axis.

## Related literature

For applications of imidazole derivatives in coordination chemistry, see: Dai & Fu (2008 ▶); Fu *et al.* (2008 ▶); Huang *et al.* (2008 ▶). 
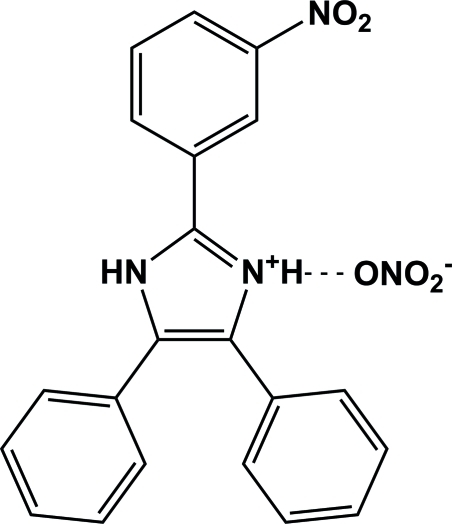


## Experimental

### 

#### Crystal data

C_21_H_16_N_3_O_2_^+^·NO_3_^−^
*M* *_r_* = 404.38
Monoclinic, 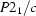

*a* = 5.870 (1) Å
*b* = 12.509 (3) Å
*c* = 26.476 (5) Å
β = 95.06 (3)°
*V* = 1936.2 (7) Å^3^
*Z* = 4
Mo *K*α radiation
μ = 0.10 mm^−1^
*T* = 298 K
0.45 × 0.40 × 0.25 mm

#### Data collection

Rigaku Mercury2 diffractometer
Absorption correction: multi-scan (*CrystalClear*; Rigaku, 2005 ▶) *T*_min_ = 0.949, *T*_max_ = 1.000 (expected range = 0.925–0.975)
19751 measured reflections
4443 independent reflections
2546 reflections with *I* > 2σ(*I*)
*R*_int_ = 0.085

#### Refinement

*R*[*F* ^2^ > 2σ(*F* ^2^)] = 0.063
*wR*(*F* ^2^) = 0.162
*S* = 1.04
4443 reflections
271 parameters
H-atom parameters constrained
Δρ_max_ = 0.19 e Å^−3^
Δρ_min_ = −0.22 e Å^−3^

### 

Data collection: *CrystalClear* (Rigaku, 2005 ▶); cell refinement: *CrystalClear*; data reduction: *CrystalClear*; program(s) used to solve structure: *SHELXS97* (Sheldrick, 2008 ▶); program(s) used to refine structure: *SHELXL97* (Sheldrick, 2008 ▶); molecular graphics: *SHELXTL* (Sheldrick, 2008 ▶); software used to prepare material for publication: *SHELXTL*.

## Supplementary Material

Crystal structure: contains datablocks I, global. DOI: 10.1107/S1600536809023599/im2120sup1.cif

Structure factors: contains datablocks I. DOI: 10.1107/S1600536809023599/im2120Isup2.hkl

Additional supplementary materials:  crystallographic information; 3D view; checkCIF report

## Figures and Tables

**Table 1:** Hydrogen-bond geometry (Å, °)

*D*—H⋯*A*	*D*—H	H⋯*A*	*D*⋯*A*	*D*—H⋯*A*
N1—H1*A*⋯O3^i^	0.86	1.93	2.768 (2)	165
N2—H2*A*⋯O3	0.86	1.88	2.705 (2)	160

Symmetry code: (i) 
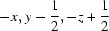
.

**Fig. 1.**
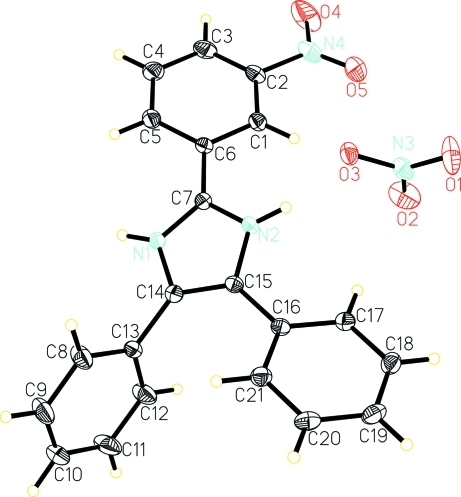
A view of the title compound with the atomic numbering scheme. Displacement ellipsoids were drawn at the 30% probability level.

**Fig. 2.**
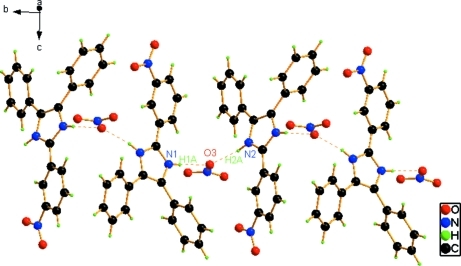
Crystal packing of the title compound viewed down a axis showing the one-dimensional chain made up by hydrogen bonding (dashed lines). Hydrogen atoms not involved in hydrogen bonding have been omitted for clarity.

## supplementary crystallographic information

### Comment

Imidazole derivatives have found wide range of applications in coordination chemistry because of their multiple coordination modes as ligands to metal ions and for the construction of novel metal-organic frameworks (Huang *et al.* 2008; Fu *et al.* 2008; Dai & Fu 2008). We report herein the crystal structure of the title compound, 2-(3-nitrophenyl)-4,5-diphenyl-1*H*-imidazol-3-ium nitrate.

The title compound contains an organic cation and a nitrate anion in the asymmetric unit. The imidazole N atom in 3-position is protonated. Benzene and imidazole rings are twisted from each other by dihedral angles of 22.75 (1)°, 79.63 (1)° and 29.65 (1)°. The crystal packing is stabilized by N—H···O hydrogen bonds to form an infinite one-dimensional chain parallel to *b* axis (Table 1, Fig. 2).

### Experimental

Under nitrogen protection, 1,2-diphenyl-ethane-1,2-dione (20 mmol), 3-nitrobenzaldehyde (20 mmol) and amine acetate (50 mmol) were dissolved in 60 ml of HOAc. The mixture was stirred at 110 °C for 20 h. The resulting solution was poured into ice water (200 ml) and after neutralizing the mixture with NaOH (6 mol/*L*) a white solid was obtained. After filtration and washing with distilled water the crude product was recrystallized from an ethanolic solution (150 ml) to which nitric acid (5 ml) was added to yield colorless block-like crystals of the title compound, suitable for X-ray analysis.

### Refinement

All H atoms attached to C and N atoms were fixed geometrically and treated as riding with C–H = 0.93 Å and N–H = 0.86 Å with *U*_iso_(H) = 1.2Ueq(C or N).

### Crystal data

**Table:**

C_21_H_16_N_3_O_2_^+^·NO_3_^−^	*F*(000) = 840
*M**_r_* = 404.38	*D*_x_ = 1.387 Mg m^−^^3^
Monoclinic, *P*2_1_/*c*	Mo *K*α radiation, λ = 0.71073 Å
Hall symbol: -P 2ybc	Cell parameters from 4443 reflections
*a* = 5.870 (1) Å	θ = 3.1–27.5°
*b* = 12.509 (3) Å	µ = 0.10 mm^−^^1^
*c* = 26.476 (5) Å	*T* = 298 K
β = 95.06 (3)°	Block, colorless
*V* = 1936.2 (7) Å^3^	0.45 × 0.40 × 0.25 mm
*Z* = 4	

### Data collection

**Table:**

Rigaku Mercury2 diffractometer	4443 independent reflections
Radiation source: fine-focus sealed tube	2546 reflections with *I* > 2σ(*I*)
graphite	*R*_int_ = 0.085
Detector resolution: 13.6612 pixels mm^-1^	θ_max_ = 27.5°, θ_min_ = 3.1°
CCD profile fitting scans	*h* = −7→7
Absorption correction: multi-scan (*CrystalClear*; Rigaku, 2005)	*k* = −16→16
*T*_min_ = 0.949, *T*_max_ = 1.000	*l* = −34→34
19751 measured reflections	

### Refinement

**Table:**

Refinement on *F*^2^	Primary atom site location: structure-invariant direct methods
Least-squares matrix: full	Secondary atom site location: difference Fourier map
*R*[*F*^2^ > 2σ(*F*^2^)] = 0.063	Hydrogen site location: inferred from neighbouring sites
*wR*(*F*^2^) = 0.162	H-atom parameters constrained
*S* = 1.03	*w* = 1/[σ^2^(*F*_o_^2^) + (0.0623*P*)^2^ + 0.3275*P*] where *P* = (*F*_o_^2^ + 2*F*_c_^2^)/3
4443 reflections	(Δ/σ)_max_ < 0.001
271 parameters	Δρ_max_ = 0.19 e Å^−^^3^
0 restraints	Δρ_min_ = −0.22 e Å^−^^3^

### Special details

**Table:**

Geometry. All e.s.d.'s (except the e.s.d. in the dihedral angle between two l.s. planes) are estimated using the full covariance matrix. The cell e.s.d.'s are taken into account individually in the estimation of e.s.d.'s in distances, angles and torsion angles; correlations between e.s.d.'s in cell parameters are only used when they are defined by crystal symmetry. An approximate (isotropic) treatment of cell e.s.d.'s is used for estimating e.s.d.'s involving l.s. planes.
Refinement. Refinement of *F*^2^ against ALL reflections. The weighted *R*-factor *wR* and goodness of fit *S* are based on *F*^2^, conventional *R*-factors *R* are based on *F*, with *F* set to zero for negative *F*^2^. The threshold expression of *F*^2^ > σ(*F*^2^) is used only for calculating *R*-factors(gt) *etc*. and is not relevant to the choice of reflections for refinement. *R*-factors based on *F*^2^ are statistically about twice as large as those based on *F*, and *R*- factors based on ALL data will be even larger.

### Fractional atomic coordinates and isotropic or equivalent isotropic displacement parameters (Å^2^)

**Table:**

	*x*	*y*	*z*	*U*_iso_*/*U*_eq_	
O3	0.2948 (3)	0.82802 (12)	0.22754 (7)	0.0478 (5)	
N3	0.5060 (4)	0.84341 (19)	0.22241 (8)	0.0512 (6)	
O2	0.6316 (4)	0.76676 (19)	0.21888 (9)	0.0876 (8)	
O1	0.5694 (4)	0.93609 (19)	0.22221 (11)	0.0982 (9)	
N2	0.1754 (3)	0.64621 (14)	0.27380 (6)	0.0341 (4)	
H2A	0.2441	0.6959	0.2586	0.041*	
N1	−0.0446 (3)	0.51473 (14)	0.28709 (7)	0.0354 (5)	
H1A	−0.1439	0.4645	0.2819	0.043*	
C7	0.0303 (4)	0.57674 (17)	0.25109 (8)	0.0332 (5)	
C13	0.0275 (4)	0.47970 (17)	0.37962 (8)	0.0356 (5)	
C16	0.3635 (4)	0.68723 (17)	0.35972 (8)	0.0347 (5)	
C1	0.1235 (4)	0.60288 (18)	0.16282 (9)	0.0416 (6)	
H1	0.2548	0.6404	0.1743	0.050*	
C18	0.7179 (4)	0.7836 (2)	0.37476 (11)	0.0490 (7)	
H18	0.8493	0.8118	0.3627	0.059*	
C15	0.2004 (4)	0.62735 (17)	0.32540 (8)	0.0337 (5)	
C2	0.0714 (5)	0.5842 (2)	0.11185 (9)	0.0472 (6)	
C21	0.3271 (4)	0.70151 (19)	0.41038 (9)	0.0440 (6)	
H21	0.1955	0.6741	0.4227	0.053*	
C6	−0.0249 (4)	0.56435 (17)	0.19669 (8)	0.0347 (5)	
C17	0.5628 (4)	0.72880 (18)	0.34219 (9)	0.0404 (6)	
H17	0.5910	0.7195	0.3085	0.049*	
C5	−0.2202 (4)	0.5095 (2)	0.17802 (9)	0.0474 (6)	
H5	−0.3215	0.4842	0.2003	0.057*	
C14	0.0604 (4)	0.54308 (17)	0.33367 (8)	0.0344 (5)	
C20	0.4843 (5)	0.7559 (2)	0.44270 (10)	0.0517 (7)	
H20	0.4578	0.7649	0.4766	0.062*	
O5	0.3866 (4)	0.6812 (2)	0.09223 (9)	0.0871 (7)	
C8	−0.1602 (5)	0.4912 (2)	0.40612 (11)	0.0607 (8)	
H8	−0.2725	0.5405	0.3953	0.073*	
N4	0.2321 (5)	0.6234 (2)	0.07626 (10)	0.0687 (7)	
C19	0.6799 (5)	0.7970 (2)	0.42501 (11)	0.0535 (7)	
H19	0.7858	0.8335	0.4468	0.064*	
C10	−0.0231 (5)	0.3584 (2)	0.46502 (10)	0.0602 (8)	
H10	−0.0386	0.3186	0.4942	0.072*	
C4	−0.2650 (5)	0.4923 (2)	0.12670 (10)	0.0581 (8)	
H4	−0.3965	0.4555	0.1147	0.070*	
O4	0.1968 (5)	0.5963 (2)	0.03177 (9)	0.1162 (10)	
C12	0.1888 (5)	0.4055 (2)	0.39616 (11)	0.0652 (9)	
H12	0.3178	0.3963	0.3786	0.078*	
C11	0.1621 (6)	0.3445 (3)	0.43847 (11)	0.0758 (10)	
H11	0.2716	0.2934	0.4489	0.091*	
C3	−0.1176 (5)	0.5290 (2)	0.09306 (10)	0.0557 (7)	
H3	−0.1459	0.5165	0.0585	0.067*	
C9	−0.1853 (5)	0.4305 (3)	0.44881 (11)	0.0700 (9)	
H9	−0.3141	0.4392	0.4665	0.084*	

### Atomic displacement parameters (Å^2^)

**Table:**

	*U*^11^	*U*^22^	*U*^33^	*U*^12^	*U*^13^	*U*^23^
O3	0.0438 (10)	0.0367 (9)	0.0637 (12)	−0.0033 (8)	0.0099 (9)	0.0069 (8)
N3	0.0560 (15)	0.0527 (14)	0.0477 (13)	0.0027 (12)	0.0209 (11)	0.0054 (11)
O2	0.0859 (17)	0.0920 (16)	0.0904 (17)	0.0467 (14)	0.0390 (14)	0.0154 (14)
O1	0.0733 (16)	0.0666 (15)	0.161 (3)	−0.0267 (12)	0.0439 (15)	0.0050 (16)
N2	0.0397 (11)	0.0327 (10)	0.0304 (10)	−0.0023 (8)	0.0057 (8)	0.0031 (8)
N1	0.0363 (10)	0.0369 (10)	0.0333 (11)	−0.0043 (8)	0.0041 (8)	0.0007 (9)
C7	0.0347 (12)	0.0315 (11)	0.0335 (12)	−0.0001 (10)	0.0037 (10)	−0.0007 (10)
C13	0.0411 (13)	0.0350 (12)	0.0310 (12)	−0.0011 (10)	0.0039 (10)	0.0015 (10)
C16	0.0391 (13)	0.0318 (12)	0.0330 (13)	0.0041 (10)	0.0024 (10)	0.0015 (10)
C1	0.0480 (14)	0.0373 (13)	0.0398 (14)	−0.0034 (11)	0.0054 (11)	0.0010 (11)
C18	0.0384 (14)	0.0482 (15)	0.0594 (18)	−0.0009 (11)	−0.0009 (12)	0.0042 (14)
C15	0.0374 (12)	0.0338 (12)	0.0303 (12)	0.0027 (10)	0.0047 (9)	0.0006 (10)
C2	0.0637 (17)	0.0449 (15)	0.0339 (14)	−0.0004 (13)	0.0091 (12)	0.0055 (12)
C21	0.0441 (14)	0.0497 (15)	0.0380 (14)	0.0005 (11)	0.0017 (11)	0.0000 (12)
C6	0.0398 (13)	0.0318 (12)	0.0321 (13)	0.0014 (10)	0.0017 (10)	0.0023 (10)
C17	0.0407 (14)	0.0415 (13)	0.0388 (13)	0.0027 (11)	0.0019 (11)	0.0017 (11)
C5	0.0505 (15)	0.0481 (15)	0.0434 (15)	−0.0081 (12)	0.0027 (12)	0.0024 (12)
C14	0.0350 (12)	0.0346 (12)	0.0340 (13)	0.0042 (10)	0.0054 (10)	−0.0003 (10)
C20	0.0612 (18)	0.0568 (17)	0.0352 (14)	0.0056 (14)	−0.0066 (13)	−0.0043 (12)
O5	0.0782 (16)	0.115 (2)	0.0699 (15)	−0.0193 (15)	0.0203 (13)	0.0227 (14)
C8	0.0539 (17)	0.0628 (18)	0.0692 (19)	0.0168 (14)	0.0262 (14)	0.0261 (16)
N4	0.092 (2)	0.0691 (17)	0.0481 (16)	0.0050 (16)	0.0231 (14)	0.0119 (14)
C19	0.0531 (17)	0.0487 (16)	0.0546 (17)	0.0003 (13)	−0.0185 (14)	−0.0015 (14)
C10	0.074 (2)	0.0654 (19)	0.0424 (16)	−0.0017 (16)	0.0105 (14)	0.0163 (14)
C4	0.0692 (19)	0.0573 (17)	0.0449 (16)	−0.0155 (14)	−0.0119 (14)	0.0000 (14)
O4	0.189 (3)	0.121 (2)	0.0462 (15)	−0.029 (2)	0.0507 (16)	−0.0077 (14)
C12	0.0593 (18)	0.087 (2)	0.0529 (18)	0.0242 (16)	0.0230 (14)	0.0262 (16)
C11	0.082 (2)	0.089 (2)	0.0580 (19)	0.0300 (19)	0.0143 (17)	0.0364 (18)
C3	0.082 (2)	0.0526 (16)	0.0315 (14)	0.0005 (15)	0.0004 (13)	−0.0021 (13)
C9	0.068 (2)	0.079 (2)	0.068 (2)	0.0061 (17)	0.0356 (16)	0.0236 (18)

### Geometric parameters (Å, °)

**Table:**

O3—N3	1.273 (3)	C2—N4	1.475 (3)
N3—O1	1.218 (3)	C21—C20	1.381 (4)
N3—O2	1.218 (3)	C21—H21	0.9300
N2—C7	1.323 (3)	C6—C5	1.389 (3)
N2—C15	1.381 (3)	C17—H17	0.9300
N2—H2A	0.8600	C5—C4	1.378 (3)
N1—C7	1.333 (3)	C5—H5	0.9300
N1—C14	1.376 (3)	C20—C19	1.377 (4)
N1—H1A	0.8600	C20—H20	0.9300
C7—C6	1.456 (3)	O5—N4	1.206 (3)
C13—C8	1.365 (3)	C8—C9	1.380 (4)
C13—C12	1.370 (3)	C8—H8	0.9300
C13—C14	1.479 (3)	N4—O4	1.226 (3)
C16—C21	1.388 (3)	C19—H19	0.9300
C16—C17	1.396 (3)	C10—C9	1.353 (4)
C16—C15	1.466 (3)	C10—C11	1.357 (4)
C1—C2	1.377 (3)	C10—H10	0.9300
C1—C6	1.390 (3)	C4—C3	1.374 (4)
C1—H1	0.9300	C4—H4	0.9300
C18—C19	1.378 (4)	C12—C11	1.376 (4)
C18—C17	1.380 (3)	C12—H12	0.9300
C18—H18	0.9300	C11—H11	0.9300
C15—C14	1.366 (3)	C3—H3	0.9300
C2—C3	1.363 (4)	C9—H9	0.9300
			
O1—N3—O2	124.2 (3)	C16—C17—H17	119.9
O1—N3—O3	116.4 (2)	C4—C5—C6	120.5 (2)
O2—N3—O3	119.4 (2)	C4—C5—H5	119.7
C7—N2—C15	110.19 (18)	C6—C5—H5	119.7
C7—N2—H2A	124.9	C15—C14—N1	106.46 (19)
C15—N2—H2A	124.9	C15—C14—C13	132.0 (2)
C7—N1—C14	109.94 (18)	N1—C14—C13	121.24 (19)
C7—N1—H1A	125.0	C19—C20—C21	120.3 (2)
C14—N1—H1A	125.0	C19—C20—H20	119.8
N2—C7—N1	107.27 (19)	C21—C20—H20	119.8
N2—C7—C6	126.7 (2)	C13—C8—C9	120.9 (3)
N1—C7—C6	125.9 (2)	C13—C8—H8	119.6
C8—C13—C12	118.2 (2)	C9—C8—H8	119.6
C8—C13—C14	122.4 (2)	O5—N4—O4	124.1 (3)
C12—C13—C14	119.3 (2)	O5—N4—C2	118.6 (3)
C21—C16—C17	118.6 (2)	O4—N4—C2	117.3 (3)
C21—C16—C15	121.1 (2)	C20—C19—C18	119.6 (2)
C17—C16—C15	120.3 (2)	C20—C19—H19	120.2
C2—C1—C6	118.4 (2)	C18—C19—H19	120.2
C2—C1—H1	120.8	C9—C10—C11	119.6 (3)
C6—C1—H1	120.8	C9—C10—H10	120.2
C19—C18—C17	120.6 (2)	C11—C10—H10	120.2
C19—C18—H18	119.7	C3—C4—C5	120.7 (3)
C17—C18—H18	119.7	C3—C4—H4	119.6
C14—C15—N2	106.10 (19)	C5—C4—H4	119.6
C14—C15—C16	131.6 (2)	C13—C12—C11	120.7 (3)
N2—C15—C16	122.16 (19)	C13—C12—H12	119.6
C3—C2—C1	123.1 (2)	C11—C12—H12	119.6
C3—C2—N4	118.8 (2)	C10—C11—C12	120.3 (3)
C1—C2—N4	118.1 (2)	C10—C11—H11	119.9
C20—C21—C16	120.7 (2)	C12—C11—H11	119.9
C20—C21—H21	119.6	C2—C3—C4	118.1 (2)
C16—C21—H21	119.6	C2—C3—H3	120.9
C5—C6—C1	119.1 (2)	C4—C3—H3	120.9
C5—C6—C7	120.6 (2)	C10—C9—C8	120.2 (3)
C1—C6—C7	120.2 (2)	C10—C9—H9	119.9
C18—C17—C16	120.2 (2)	C8—C9—H9	119.9
C18—C17—H17	119.9		

### Hydrogen-bond geometry (Å, °)

**Table:**

*D*—H···*A*	*D*—H	H···*A*	*D*···*A*	*D*—H···*A*
N1—H1A···O3^i^	0.86	1.93	2.768 (2)	165
N2—H2A···O3	0.86	1.88	2.705 (2)	160

Symmetry codes: (i) −*x*, *y*−1/2, −*z*+1/2.
